# Electrochemotherapy of cutaneous metastastes from breast cancer in elderly patients: a preliminary report

**DOI:** 10.1186/1471-2482-12-S1-S6

**Published:** 2012-11-15

**Authors:** Raffaella Benevento, Antonio Santoriello, Giuseppe Perna, Silvestro Canonico

**Affiliations:** 1 Department of Medical, Surgical, Neurologic, Metabolic and Ageing Sciences, Second University of Naples, Naples, Italy

## Abstract

**Background:**

The management of cutaneous metastases often represents a challenge because they may be widespread and may recur after radiotherapy or chemotherapy; breast cancer accounts for 51% of the total cases of cutaneous metastases. When surgical excision of chest wall recurrences is not possible and other local treatments such as radiotherapy or radiotherapy with hyperthermia fail, topical chemotherapy and electrochemotherapy (ECT) might be taken into account.

ECT is a new local treatment of solid tumors which can be defined as the local potentiation, by means of permeabilizing electric pulses, of the antitumor activity of a non permeating anticancer drug with high intrinsic cytotoxicity.

**Methods:**

This prospective observational study took place throughout March 2010 to October 2011. Twelve consecutive elderly patients (1 man and 11 women, median age of 76 years) with regional or distant skin or subcutaneous metastases from breast cancer, with or without visceral disease, were included in the study. Patient enrollment was carried out according to the ESOPE criteria. Bleomycin administration was followed by the application of brief electric pulses to each tumor nodule within 8 min after intravenous infusion of the drug. Electric currents were delivered by means of a 2–3 cm long needle electrode according to lesion size. All treatments were performed using the Cliniporator^TM^ device.

**Results:**

We observed Complete Response(CR) in 75.3% (107 metastases), Partial Response(PR) in 17% (24 metastases), no change in 7.7% (11 metastases) . No serious ECT-related adverse events were reported; adverse events consisted of pain in the treated area one to two days after treatment (1 patient, 8.3%) and ulceration of treated area (1 patient, 8.3%).

**Conclusion:**

ECT could be suggested as a primary local therapy in patients not suitable for surgical removal of the primary tumor, and clinicians should not hesitate to use it even in the elderly.

## Background

Skin metastases from breast cancer are relatively common and sometimes impair quality of life. Review of the literature indicates that the incidence of cutaneous metastases for all types of carcinomas ranges from 0.7% to 10.0% [1][2]. Breast cancer accounts for 51% of the total cases of cutaneous metastases [1,2].

The management of cutaneous metastases often represents a challenge for the clinician, and surgical excision might be followed by radiotherapy and systemic treatment. When surgical excision is not possible there are several therapeutic options such as radiotherapy, systemic chemo- and polychemotherapy, laser ablation, or radiofrequency ablation [3-5] .Several recent clinical studies proposed electrochemotherapy (ECT) as a novel, complementary therapeutic weapon for controlling cutaneous and subcutaneous metastases [6,7]. ECT is a non-thermal tumor ablation modality, based on electroporation. The principle of the method is the application of electric currents (electric pulses) to the cancer cells using externally applied electrical fields, that increase cell membrane permeability transiently, and enhance the penetration of drugs into cancer cells, thus facilitating transportation of the chemotherapeutics into the cancer cells [8,9].

Bleomycin and cisplatin have proven to be the most suitable candidates for the combined use with electroporation based on their chemical properties and molecular intracellular targets. Association with electroporation induces a cytotoxicity increase of up to 80-fold for cisplatin and up to 8000-fold for bleomycin [10].

The required dose of bleomycin is 15.000 IU/m2 with intravenous administration. The application of the electric pulses without drugs has minimal or no effect on tumor growth [11-13].

ECT was originally employed for the treatment of metastatic head and neck cancer and has been used in the treatment of cutaneous metastases from tumours independent of histology [14,15].

## Methods

This prospective, observational study took place throughout March 2010 to October 2011. Twelve consecutive elderly patients (1 man and 11 women) with a median age of 76 years, with regional or distant skin or subcutaneous metastases from breast cancer, with or without visceral disease, were included in the study. Inclusion criteria were as follows: the tumors were unsuitable for resection or radiotherapy; they were unresponsive to, or progressed after, two or three lines of systemic chemotherapy; the patient Karnofsky performance status was greater than 70%.

Exclusion criteria were: coagulation disorder( A platelet count < 50000/ml was required, with a prothrombin time < 40 s and an activated partial thromboplastin time in the normal range); active infection; progressive visceral disease or allergy to bleomycin; severe renal or hepatic insufficiency; short life expectancy (<3 months).

Patient enrollment was carried out according to the ESOPE criteria [6].

The anesthesiologic technique was a mild general anesthesia for all the procedures. Bleomycin administration was followed (Bleomycin Nippon-Kayaku, Sanofi-Aventis, vials 15 mg, 15,000 UI/m2) by the application of brief electric pulses to each tumor nodule within 8 min after intravenous infusion of the drug. Electric currents were delivered by means of a 2–3-cm long needle electrode according to lesion size. All treatments were performed using the Cliniporator^TM^ device (IGEA Ltd., Modena, Italy). For best results, the drug must be administered rapidly and the electroporation must begin within 8 min of the complete administration of the drug and is to be completed in 25 min.

## Results

Twelve patients with 142 nodules were treated in one-day surgery with ECT. The diameter of cutaneous and subcutaneous metastases was 0.5-1 cm in 7 patiens, (58.3%), 1,5-2,5 cm in 3 patients (25%), > 3 cm in 2 patients (16.7%).

The median observation time of treated metastases was 210 days (range 30–354 days). Response rate was determined at least 30 days after ECT in each case. The response rate was evaluated according to the Response Evaluation Criteria in Solid Tumours (RECIST version 1.0) [16].

We observed Complete Response (CR) in 75.3% (107 metastases), Partial Response (PR) in 17% (24 metastases), no change in 7.7% (11 metastases). No serious ECT-related adverse events were reported; adverse events consisted of pain in the treated area one to two days after treatment (1 patient, 8.3%) and ulceration of treated area (1 patient, 8.3%). One patients developed dissociative amnesia and agitation that was treated with antipsychotic drug (i.m. clorpromazine).

Eight patients received a single ECT session (66.6%). A second treatment was performed in 4 patients (33.3%); one of them underwent a third ECT treatment. These four patients underwent a second session to treat the nodules that remained after the first course; one patients underwent a third treatment for both new and previously treated lesions and always maintained a PR with respect to the initial measurement. A second ECT course to treat new lesions was performed after a median of 3 (range 2–9) months after the first treatment.

## Discussion

Cutaneous metastases or recurrent malignant disease in the skin are often difficult to manage. Uncontrolled cutaneous metastases can, in many ways, adversely affect self-esteem and body image. Management of metastatic breast cancer in women over the age of 65 follows the same principles that guide treatment of younger women, with few exceptions.

Important issues in older cancer patients such as life expectancy, comorbidity, and functional status must be considered when making treatment decisions. Clinical decision-making, particularly when cytotoxic chemotherapy is being considered, requires consideration of whether an individual's life expectancy is long enough to obtain benefit from therapy and whether treatment will be adequately tolerated.

ECT can be used where surgery is not an option and is also efficient in chemotherapy-resistant and radiotherapy-resistant lesions [16,17]. Treatment may provide palliation particularly where there is pain or bleeding from cutaneous metastases [18].

It is well tolerated with few side-effects, allows for immediate recovery and can be repeated. Several clinical studies on ECT were published, demonstrating its efficacy in local tumor control of various malignancies. In ESOPE [6] study 16 patients were treated over 75 years with significant benefit to them, without side-effects. Another advantage of ECT is that it is easy and quick to perform (median time of treatment < 25 min). The treated tumor nodules are well controlled, and after complete regression of the treated nodules good cosmetic effect is obtained. ECT is a viable option in the treatment of patients with tumors unsuitable for other therapies, even if only for palliation, improving the quality of life. ECT is simple, highly effective and safe. The absence of systemic adverse effects and the low impact on immune system functions also allow the treatment of elderly people with repeated courses.

## Conclusion

Our results are in agreement with previous studies, suggesting ECT as an effective and safe loco-regional therapy for breast cancer. ECT may be an alternative treatment modality to conventional therapies, especially in case of multiple cutaneous and subcutaneous lesions. It has been proven to relief symptoms of loco-regional relapse and to improve patients’ quality of life.

Furthermore, ECT also has the potential of being advocated for the treatment of other clinical conditions, such as primary local therapy in frail elderly patients that are not suitable for surgical removal of breast cancer.

## List of Abbreviations used

ECT: Electrochemotherapy; RECIST: Response Evaluation Criteria in Solid Tumours; CR: Complete Response; PR: Partial Response.

## Competing interests

Authors have no competing interests to disclose.

## Authors’ contribution

RB designed the study, and wrote the draft of the manuscript. AS and GP collected data and participated in the design of the study. SC conceived of the study, and participated in its design and coordination and helped to draft the manuscript. All authors read and approved the final manuscript.

## References

[B1] Lookingbill DP Spanger N Helm KF Cutaneous metastases in patients with monastic carcinoma: a retrospective study of 4020 patients J Am Acad Dermatol 1993 29 228 236 10.1016/0190-9622(93)70173-Q 8335743

[B2] Spencer PS Helm TN Skin metastases in cancer patients Cutis 1987 39 119 121 3829718

[B3] Jagsi R Pierce L Postmastectomy radiation therapy for patients with locally advanced breast cancer Semin Radiat Oncol 2009 19 236 243 10.1016/j.semradonc.2009.05.009 19732688

[B4] Zagar TM Higgins KA Miles EF Vujaskovic Z Dewhirst MW Clough RW Durable palliation of breast cancer chest wall recurrence with radiation therapy, hyperthermia, and chemotherapy Radiother Oncol 2010 97 535 540 10.1016/j.radonc.2010.10.020 21074876 PMC3278216

[B5] Leonard R Hardy J van Tienhoven G Houston S Simmonds P David M Randomized, double-blind, placebo-controlled, multicenter trial of 6% miltefosine solution, a topical chemotherapy in cutaneous metastases from breast cancer J Clin Oncol 2001 21 4150 4159 11689583 10.1200/JCO.2001.19.21.4150

[B6] Marty M Sersa G Garbay JR Gehl J Electrochemotherapy an easy, highly effective and safe treatment of cutaneous and subcutaneous metastases: results of ESOPE (European Standard Operating Procedures of Electrochemotherapy) study EJC Suppl 2006 4 3 13

[B7] Campana LG Mocellin S Basso M Puccetti O Bleomycinbased electrochemotherapy: clinical outcome from a single institution’s experience with 52 patients Ann Surg Oncol 2009 16 191 199 10.1245/s10434-008-0204-8 18987914

[B8] Neumann E Schaeferridder M Wang Y Hofschneider P Gene-transfer into mouse lyoma cells by electroporation in high electric-fields EMBO J 1982 1 841 845 6329708 10.1002/j.1460-2075.1982.tb01257.x PMC553119

[B9] Mir LM Orlowski S Mechanisms of electrochemotherapy Adv Drug Deliv Rev 1999 35 107 118 10.1016/S0169-409X(98)00066-0 10837692

[B10] Orlowski S Belehradek J Jr Paoletti C Transient electropermeabilization of cells in culture. Increase of the cytotoxicity of anticancer drugs Biochem Pharmacol 1988 37 4727 4733 10.1016/0006-2952(88)90344-9 2462423

[B11] Sersa G Cemazar M Miklavcic D Antitumor effectiveness of electrochemotherapy with cis-diamminedichloroplatinum(II) in mice Cancer Res 1995 55 3450 3455 7614485

[B12] Mir LM Orlowski S Belehradek J Jr Paoletti C Electrochemotherapy potentiation of antitumour effect of bleomycin by local electric pulses Eur J Cancer 1991 27 68 72 10.1016/0277-5379(91)90064-K 1707289

[B13] Belehradek J Jr Orlowski S Poddevin B Paoletti C Electrochemotherapy of spontaneous mammary tumours in mice Eur J Cancer 1991 27 73 76 10.1016/0277-5379(91)90065-L 1707290

[B14] Snoj M Cemazar M Slekovec Kolar B Sersa G Effective treatment of multiple unresectable skin melanoma metastases by electrochemotherapy: case report Croat Med J 2007 48 345 349 PMC2080549 17589984

[B15] Belehradek M Domenge C Luboinski B Orlowski S Belehradek J Jr. Mir LM Electrochemotherapy, a new antitumor treatment. First clinical phase I-II trial Cancer 1993 72 3694 3700 10.1002/1097-0142(19931215)72:12<3694::AID-CNCR2820721222>3.0.CO;2-2 7504576

[B16] Therasse P Arbuck SG Eisenhauer EA New guidelines to evaluate the response to treatment in solid tumors. European Organization for Research and Treatment of Cancer, National Cancer Institute of the United States, National Cancer Institute of Canada J Natl Cancer Inst 2000 92 205 216 10.1093/jnci/92.3.205 10655437

[B17] Rodriguez-Cuevas S Barroso-Bravo S Manza-Estrada J Cristobal-Martinez L Gonzalez-Rodriguez E Electrochemotherapy in primary and metastatic skin tumors: phase II trial using intralesional bleomycin Arch Med Res 2001 32 273 276 10.1016/S0188-4409(01)00278-8 11440782

[B18] Gothelf A Mir LM Gehl J Electrochemotherapy: results of cancer treatment using enhanced delivery of bleomycin by electroporation Cancer Treat Rev 2003 29 371 387 10.1016/S0305-7372(03)00073-2 12972356

